# The miR-590-3p/CFHR3/STAT3 signaling pathway promotes cell proliferation and metastasis in hepatocellular carcinoma

**DOI:** 10.18632/aging.204178

**Published:** 2022-07-18

**Authors:** Zhongzhong Wan, Xingrun Li, Xinru Luo, Bofan Wang, Xiang Zhou, Ao Chen

**Affiliations:** 1 Institute of Biology and Medicine, College of Life Science and Health, Wuhan University of Science and Technology, Wuhan 430081, Hubei Province, People’s Republic of China; 2 State Key Laboratory for Liver Research, The University of Hong Kong, Hong Kong 0000, People’s Republic of China; 3 Department of Pathology, Li Ka Shing Faculty of Medicine, The University of Hong Kong, Hong Kong 0000, People’s Republic of China

**Keywords:** miR-590-3p, CFHR3, STAT3 phosphorylation, S3I-201, HCC

## Abstract

Accumulating evidence has indicated that Complement factor H-related 3 (CFHR3) plays an essential role in various diseases. However, the biological functions of CFHR3 in hepatocellular carcinoma (HCC) remain largely unclear. Therefore, we perform a further study on CFHR3 in HCC. In this article, we report the suppressive role of CFHR3 in the proliferation and metastasis of HCC cells. CFHR3 downregulation is closely associated with large (T3-T4) HCC, tumor recurrence, and advanced (stage III-IV) clinical stage, functioning as an independent factor for the prognoses of HCC patients. Knockdown of CFHR3 promotes proliferation, migration, and invasion of HCC cells. Mechanistically, downregulation of CFHR3 is induced by miR-590-3p binding to the 3’ untranslated region (UTR) of CFHR3. CFHR3 downregulation promotes the phosphorylation of STAT3 protein, thereby suppressing p53 expression. The promotional effect upon downregulation of CFHR3 induced by CFHR3 stable knockdown or miR-590-3p on HCC cell malignant phenotypes is attenuated by STAT3 inhibitor, S3I-201. In conclusion, our results reveal that CFHR3 is a protective biomarker for HCC patients, and targeting the miR-590-3p/CFHR3/p-STAT3/p53 signaling axis provides a promising strategy for HCC therapeutics.

## INTRODUCTION

Hepatocellular carcinoma (HCC), the fifth most common malignant tumor, prevails with more than 700,000 new cases and 750,000 deaths globally every year [1, 2]. Although the progress in surgery has significantly increased the survival rate of HCC patients at the early stage, a large proportion of patients were diagnosed at the advanced stage due to the rapid development and progression, and less than 30% of patients in the initial diagnosis were available for surgical resection, resulting in a low 5-year survival rate [3]. With these concerns, it is urgently needed to identify more novel therapeutic molecular biomarkers to develop a more effective treatment strategy for HCC patients.

The human complement factor H-related 3 (CFHR3) is a family member of the complement factor H-related (CFH) proteins [4]. CFH proteins have been reported to be associated with some natural diseases including age-related macular degeneration (AMD), atypical hemolytic uremic syndrome (aHUS) as well as multiple cancers [5–7]. These related diseases are mainly regarded as the induction by the homozygous/heterozygous loss-of-function mutations in CFH genes, and the deletion of CFHR1 or CFHR3 [8–10]. Previous reports have indicated that cancer cells escape from immune surveillance by utilizing CFH proteins [11–13]. In addition, abnormal expression of CFHR3 was shown to be related to chemoresistance in gliomas [14]. However, little is known about the relationship between CFHR3 and the malignant phenotypes of HCC.

In our study, the mRNA levels of CFHR3 with the clinical implications were firstly analyzed based on the TCGA-LIHC database. The aggressive characteristics of CFHR3 downregulation in HCC cell biological behaviors were revealed. Further, we found that reduction of CFHR3 in HCC resulted from miR-590-3p overexpression, promoting phosphorylation of STAT3, suppressing p53 to promote the proliferation and metastasis in HCC cells, which is constricted by STAT3 inhibitor, S3I-201. Taken together, these data indicate a molecular mechanism that CFHR3 downregulation induced by miR-590-3p, promotes HCC malignant phenotypes through facilitating phosphorylation of STAT3.

## MATERIALS AND METHODS

### Cell lines

The HCC cells including PLC/PRF/5, Huh7, HepG2, Hep3B, and MHCC-97L cells, as well as immortalized human hepatocytes (MIHA cells), were obtained from the Procell Life Science and Technology Company in China. Cells were seeded in culture plates with the RPMI 1640 medium (HyClone, USA) with 10% (v/v) fetal bovine serum (FBS) (Gibco, USA) at a humid incubator (37° C and 5% CO_2_). The cells were trypsinized by using 0.25% trypsin (Gibco, USA) and sub-cultured when cells reached 70-80% confluence. For the establishment of HCC cell lines with the stable knockdown/overexpression of corresponding genes, indicated constructs were induced into HCC cells through lentiviral infection. After that, cells were screened by adding 3 μg/ml puromycin or 5 μg/ml blasticidin.

### Constructs

For the gene knockdown, the shRNAs of CFHR3 were inserted into pLKO.1-puro vector and the target sequences along with the control sequences were exhibited in the Supplementary Materials. For the CFHR3 stable overexpression, the coding sequence of CFHR3 was inserted into the pLenti-CMV-blast vector, and the corresponding primers for cloning were shown in the Supplementary Materials. The pLKO.1-puro vector (#8453), pLenti-CMV-blast vector (#17486), and STAT3 stable overexpression plasmid (#71450) were purchased from the Addgene company (USA). In the luciferase reporter assay, the wild-type (WT) and correspondingly mutated (MUT) 3’ UTR of CFHR3 were amplified and inserted into the pGL3-Basic vector (#E1751) which was obtained from the Promega company (USA), and the information of primers was also displayed in the Supplementary Materials.

### Western blotting

Proteins were extracted from experimental cells in the RIPA lysis buffer (Invitrogen, USA) by 10-minute centrifugation at 18,000 × g at 4° C. Proteins (20 μg/lane) were separated by using SDS-PAGE (10% to 15%) and were then transferred onto the PVDF membranes, which were blocked by 5% (w/v) milk mixed in Tris-buffered saline (TBS) with 0.2% (v/v) Tween-20 (TBST) for 2 hours at room temperature (RT). Membranes with proteins were incubated with the indicated primary antibodies: CFHR3 (16583-1-AP, Proteintech, USA), JAK1 (ab133666, Abcam, UK), p-JAK1 (ab138005, Abcam, UK), STAT3 (ab68153, Abcam, UK), p-STAT3 (ab267373, Abcam, UK), p53 (ab26, Abcam, UK) and β-actin (sc-81178, Santa Cruz, USA) at 4° C overnight. After that, membranes were washed with 1× TBST three times and incubated with corresponding secondary antibodies at RT for 1.5 hours. The protein bands then were visualized and analyzed using an ECL system (Amersham, USA).

### miRNA quantitation

The total cellular RNA was extracted using the RNeasy Mini kit (#74104) which was purchased from the QIAGEN company (USA). Extracted miRNA was then reversely transcribed into cDNA by the first-strand cDNA synthesis kit (B532451-0020) which was purchased from the Shanghai Sangon Biotechnology company in China. Real-time PCR was performed on the Applied Biosystems® 7500 instrument (USA). Primers for miR-590-3p detection were listed in the Supplementary Materials.

### Cell growth assays

In brief, CFHR3 stable knockdown or overexpression HCC cells (1×10^4^) were seeded in 24-well culture plates for 5-day culture. Cells were counted and recorded at 24, 48, 72, 96, and 120 hours (N=3 at least).

### Migration and invasion assays

Briefly, cell migration and invasion abilities were determined in transwell chambers with 8-μm pore membranes (Becton Dickinson, USA) as previously described [15]. Cells fixed on the undersurface of the membranes were stained with 5% crystal violet, and then counted using a microscope (N=3 at least).

### Oligonucleotide transfection and luciferase assay

miRNA-590-3p precursors were transferred into cells by using the Lipofectamine RNAiMAX transfection kit (Invitrogen, USA). The indicated reporter vectors (CFHR3-3’ UTR and CFHR3-3’ UTR MUT vectors) together with miRNA mimics mix were transfected using Lipo2000 (Invitrogen, USA); after transfection for 48 hours, cells were harvested to analyze. The pBIND vector (#E2440, Promega, USA) was used as the internal reference; the renilla luciferase signal was used for normalizing the firefly luciferase signal. Luciferase activity was determined using the dual-luciferase reporter system (Promega, USA) for 3 trials at least.

### Animal experiments

5-week-old male NOD-SCID mice were obtained from Charles River Laboratories in Beijing in China. Mice were divided into the experimental group by researchers with random allocation. For the *in vivo* lung metastasis assays, luciferase-labeled MHCC-97L cells transfected with shCFHR3-RNAs or miR-590-3p precursor were treated with S3I-201 (50μM) or DMSO (control) and injected (2 × 10^6^ cells in 200 μL 1× PBS) into the mice in each group via tail veins. 6 weeks after the injection, the metastatic foci were visualized and analyzed.

### Bioinformatics analysis

Level-3 transcriptomic data were obtained from the TCGA platform (https://portal.gdc.cancer.gov/). The liver cancer dataset harbors the RNA sequencing data from 370 tumors together with 50 non-tumor samples. The “edgeR” package was used for calculating the gene expression levels. Candidate genes were preliminarily screened and validated by the GEPIA2 online database as previous description [16, 17]. The top 500 genes related to the prognoses of HCC patients were figured out by using the “Most Differential Survival Genes” module on the GEPIA2 website. The median of gene expression levels was used as the expression cut-off for Kaplan-Meier analyses in OS/DFS. The gene enrichments upon CFHR3 levels in HCC were analyzed by the Gene Set Enrichment Analysis (GSEA) program [18]. The miRNA bindings of the CFHR3 3’UTR region were analyzed by the TargetScan program [19].

### Statistical analysis

Version-8 GraphPad Prism software (USA) was used for statistical analyses. Pearson’s correlation test was used to investigate the relationship between indicated genes. Two-tailed independent Student’s t-test, as well as Mann-Whitney U-test, was used for comparing the differences between the two groups. Survival curves were figured out by Kaplan-Meier log-rank analyses. The data were shown as mean ± SD. Statistical significances of results were exhibited at *p < 0.05, **p < 0.01, ***p < 0.001 and ****p < 0.0001.

### Ethics approval and consent to participate

This study was reviewed and approved by the Ethics Committees of Wuhan University of Science and Technology (Wuhan, China). The study was conducted by the Institutional Animal Care and Use Committee.

## RESULTS

### CFHR3 downregulation is correlated with HCC patients’ poor prognoses

**Figure 1:**
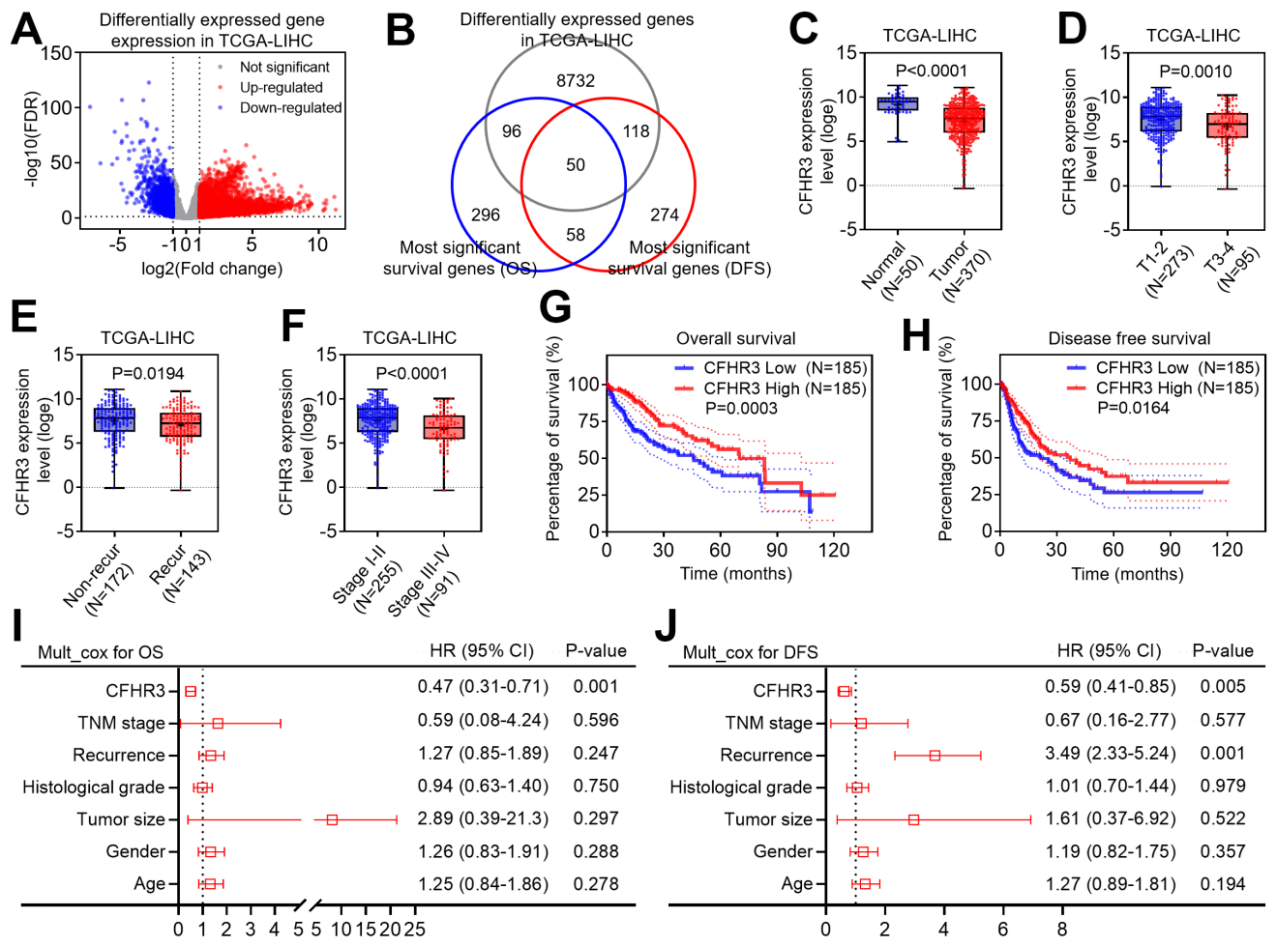
**Downregulation of CFHR3 is associated with a poor prognosis of HCC patients.** (**A**) Differentially expressed genes (DEGs) between HCC and normal liver tissues in TCGA-LIHC cohort are shown as a volcano plot. (**B**) The overlap between DEGs, the most significant overall survival (OS) related genes, and the most significantly disease-free survival (DFS) related genes in the TCGA database is displayed as a Venn diagram. (**C**) The difference in CFHR3 transcript levels between normal liver and HCC tissues is presented as a box plot. (**D**–**F**) The difference in CFHR3 transcript levels between HCC subgroups, including tumor size (**D**, T1-2, and T3-4), tumor recurrence (**E**, non-recurrence and recurrence), and clinical stage (**F**, stage I-II, and stage III-IV) is presented as a box plot, respectively. (**G**, **H**) The difference in OS rates (**G**) or DFS rates (**H**) between HCC patients with low and high CFHR3 transcript levels in HCC tissues is shown as a survival curve, respectively. (**I**, **J**) The independent prognostic factor for OS (**I**) and DFS (**J**) of HCC patients, based on the multivariate Cox proportional hazard model, is shown in each forest plot, respectively.

To clarify a potentially essential gene for HCC malignant phenotypes, we first compared the transcriptome data between HCC and normal liver tissues from the liver cancer dataset in the TCGA database, and differentially expressed gene (DEG) screening in HCC tissues was conducted based on the following selection criteria: False discovery rate (FDR) less than 0.05 and fold change larger than 2. After that, we figured out that 8996 genes significantly altered in HCC when compared to normal liver tissues (Figure 1A). The most significant OS-associated genes and DFS-associated genes were figured out by the GEPIA2 website. When overlapping the DEGs with the survival-associated genes in HCC, we finally got that 50 differentially expressed genes may be associated with HCC patients’ prognoses, and CFHR3 is one of them, which is worthy to be studied (Figure 1B). Further, it showed that in addition to low expression of CFHR3 in HCC tissues (Figure 1C), CFHR3 mRNA levels were much lower in aggressive HCC subtypes, such as large HCC (Figure 1D), recurrent HCC cases (Figure 1E), and advanced HCC cases (Figure 1F). Chi-square test analysis showed that downregulation of CFHR3 was positively relevant to elevated level alpha-fetoprotein (AFP), high histological grade, large tumor, HCC recurrence as well as advanced TNM stage (Supplementary Table 1). Kaplan-Meier survival analyses revealed that HCC patients with CFHR3^low^ trended to have the significantly higher OS and DFS rates (Figure 1G, 1H). Moreover, the multivariate Cox regression models showed that CFHR3 is an independent protective factor for HCC patients (Figure 1I, 1J). Collectively, we indicate that CFHR3 is downregulated in HCC, which is relevant to aggressive clinicopathological features and HCC patients’ poor prognoses.

### CFHR3 downregulation promotes HCC cell malignant phenotypes

**Figure 2:**
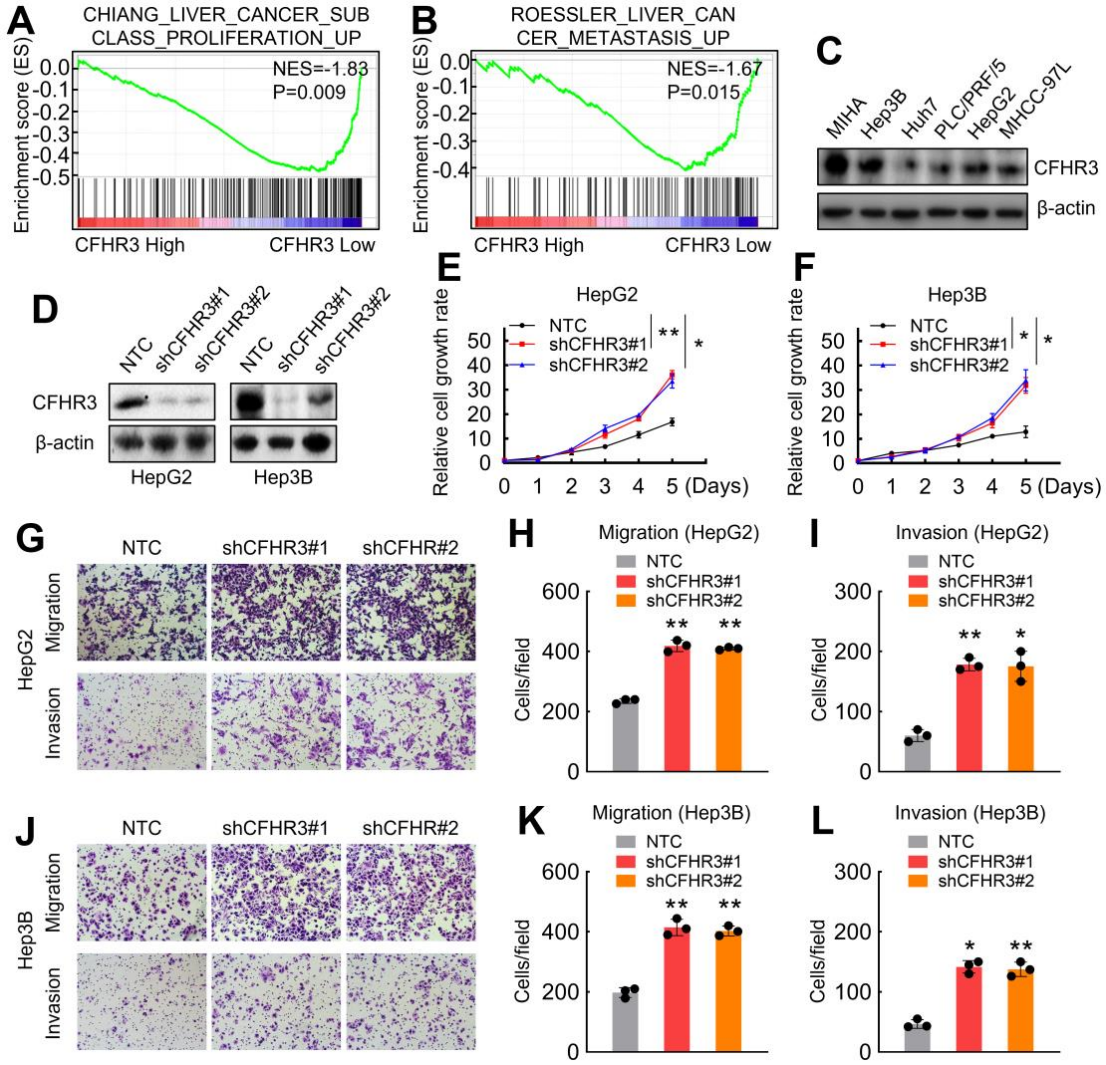
**CFHR3 downregulation promotes HCC cell malignant phenotypes.** (**A**, **B**) GSEA plots show enrichment of gene signatures associated with the liver cancer proliferation and metastasis in HCC tissues with a CFHR3 high as compared with a CFHR3 low (**A**, Chiang Liver Cancer Subclass Proliferation Up; **B**, Roessler Liver Cancer Metastasis Up). (**C**) CFHR3 protein levels in five HCC cell lines and normal liver cells (MIHA) were determined by western blot. (**D**) CFHR3 knockdown efficiency was validated by western blot in HepG2 and Hep3B cells, respectively. (**E**, **F**) Cell growth of CFHR3-knockdown HepG2 (**E**) and Hep3B (**F**) cells and negative control (NTC) cells were determined, respectively. (**G**–**L**) Migration and invasion of CFHR3-knockdown HepG2 (**G**–**I**) and Hep3B (**J**–**L**) cells and NTC cells were determined, respectively. (Remarks: * represents P < 0.05; ** represents P < 0.01; Data are presented as mean ± SD).

To demonstrate the biological roles of CFHR3 in HCC progression, functionally related gene signatures upon CFHR3 expression levels in HCC were analyzed. The GSEA results revealed that liver cancer proliferation and metastasis-related gene signatures, such as “Chiang Liver Cancer Subclass Proliferation Up” and “Roessler Liver Cancer Metastasis Up”, were enriched in HCC cases with CFHR3^Low^ (Figure 2A, 2B). To further validate the roles of CFHR3 in HCC cell growth and metastasis, CFHR3 levels were investigated in different HCC cells and normal hepatocytes (MIHA cells). It showed that CFHR3 was highly expressed in two HCC cell lines (HepG2 and Hep3B) and the MIHA cells, and it was low expressed in Huh7, PLC/PRF/5, and MHCC-97L cells (Figure 2C). Cell functional assays showed that Huh7, PLC/PRF/5 and MHCC-97L cells harbor higher capabilities of cell growth and migration (Supplementary Figure 1A–1C), indicating that CFHR3 is a crucial factor inhibiting HCC progression. Furthermore, we conducted CFHR3 knockdown in HCC cell lines (HepG2 and Hep3B) as well as the MIHA cells who harbor relatively high CFHR3 levels (Figure 2D and Supplementary Figure 1D). We further found that knockdown of CFHR3 stimulated HCC cell growth, migration as well as invasion (Figure 2E–2L). In addition, CFHR3 knockdown also promoted MIHA cell proliferation and metastasis (Supplementary Figure 1E–1G). To reconfirm the role of CFHR3 in HCC progress, we overexpressed CFHR3 in CFHR3-low HCC cells (Huh7 and PLC/PRF/5). It showed that CFHR3 overexpression exhibited the reversed phenomenon in cell proliferation and metastasis (Supplementary Figure 1H–1O). Altogether, these results demonstrate that CFHR3 inhibits HCC cell malignant phenotypes.

### CFHR3 is a critical effector for miR-590-3p, regulating HCC cell malignant phenotypes

**Figure 3:**
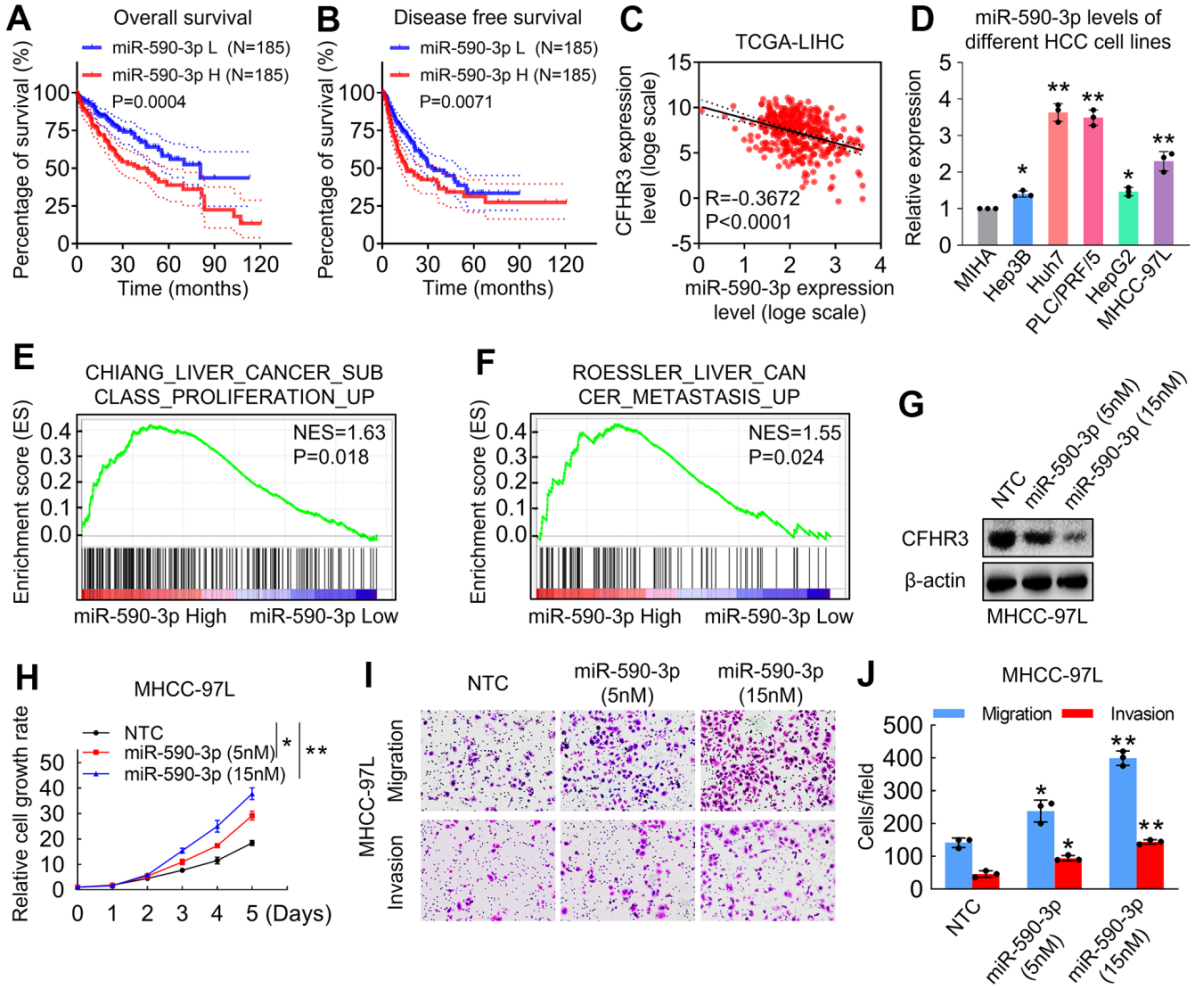
**CFHR3 is a critical effector for miR-590-3p in the regulation of HCC cell malignant phenotypes.** (**A**, **B**) The difference for OS rates (**A**) or DFS rates (**B**) between HCC patients with low and high miR-590-3p levels in HCC tissues is shown as a survival curve, respectively. (**C**) An analysis shows linear regressions and Pearson correlations between CFHR3 and miR-590-3p levels in HCC tissues in the TCGA database. (**D**) The levels of miR-590-3p in five HCC cell lines and normal liver cells (MIHA) were determined by qPCR. (**E**, **F**) GSEA plots show enrichment of gene signatures associated with the liver cancer proliferation and metastasis in HCC tissues with a miR-590-3p high as compared with miR-590-3p low (**E**, Chiang Liver Cancer Subclass Proliferation Up; **F**, Roessler Liver Cancer Metastasis Up). (**G**–**J**) MHCC-97L cells were treated with the indicated concentration of miR-590-3p precursor, and CFHR3 protein levels (**G**), cell growth (**H**), migration, and invasion (**I**, **J**) were determined. (Remarks: * represents P < 0.05; ** represents P < 0.01; Data are presented as mean ± SD).

The molecular mechanisms of downregulation of CFHR3 in HCC were further explored. We noticed that as one of the 50 candidates that we screened out, miR-590-3p upregulated HCC cases showed significantly lower OS and DFS rates (Figure 3A, 3B). As we all know, miRNAs take part in RNA silencing and downregulation of gene expression [20]. Because levels of miR-590-3p showed an opposite role in OS and DFS as compared to CFHR3 in HCC cases, we first determined the association between CFHR3 and miR-590-3p, and it showed that the levels of CFHR3 were negatively correlated with the levels of miR-590-3p in TCGA-LIHC dataset (R=-0.3672, p<0.0001; Figure 3C). Also, miR-590-3p was highly expressed in CFHR3-low HCC cells (Huh7, PLC/PRF/5, and MHCC-97L) and was low expressed in CFHR3-high HCC cells (HepG2 and Hep3B) and MIHA cells (Figure 3D). In addition, liver cancer proliferation and metastasis-related gene signatures were shown to be enriched in HCC tissues with high miR-590-3p expression (Figure 3E, 3F). Furthermore, we selected the MHCC-97L cells that have moderate and appropriate levels of CFHR3 and miR-590-3p among the five different HCC cell lines (Figures 2C, 3D) for mechanism study in HCC. It indicated that miR-590-3p suppressed CFHR3 in a dose-dependent manner in HCC cells (Figure 3G). More important, miR-590-3p enhanced HCC cell proliferation, migration as well as invasion in a dose-dependent manner (Figure 3H–3J). Except for the HCC cells, miR-590-3p also promoted the cell growth, migration, and invasion of MIHA cells (Supplementary Figure 2A–2D). Collectively, these results suggest that CFHR3 is a potential effector for miR-590-3p regulating HCC cell malignant phenotypes.

### Downregulation of CFHR3 is caused by the binding of miR-590-3p with the 3’ UTR of CFHR3

**Figure 4:**
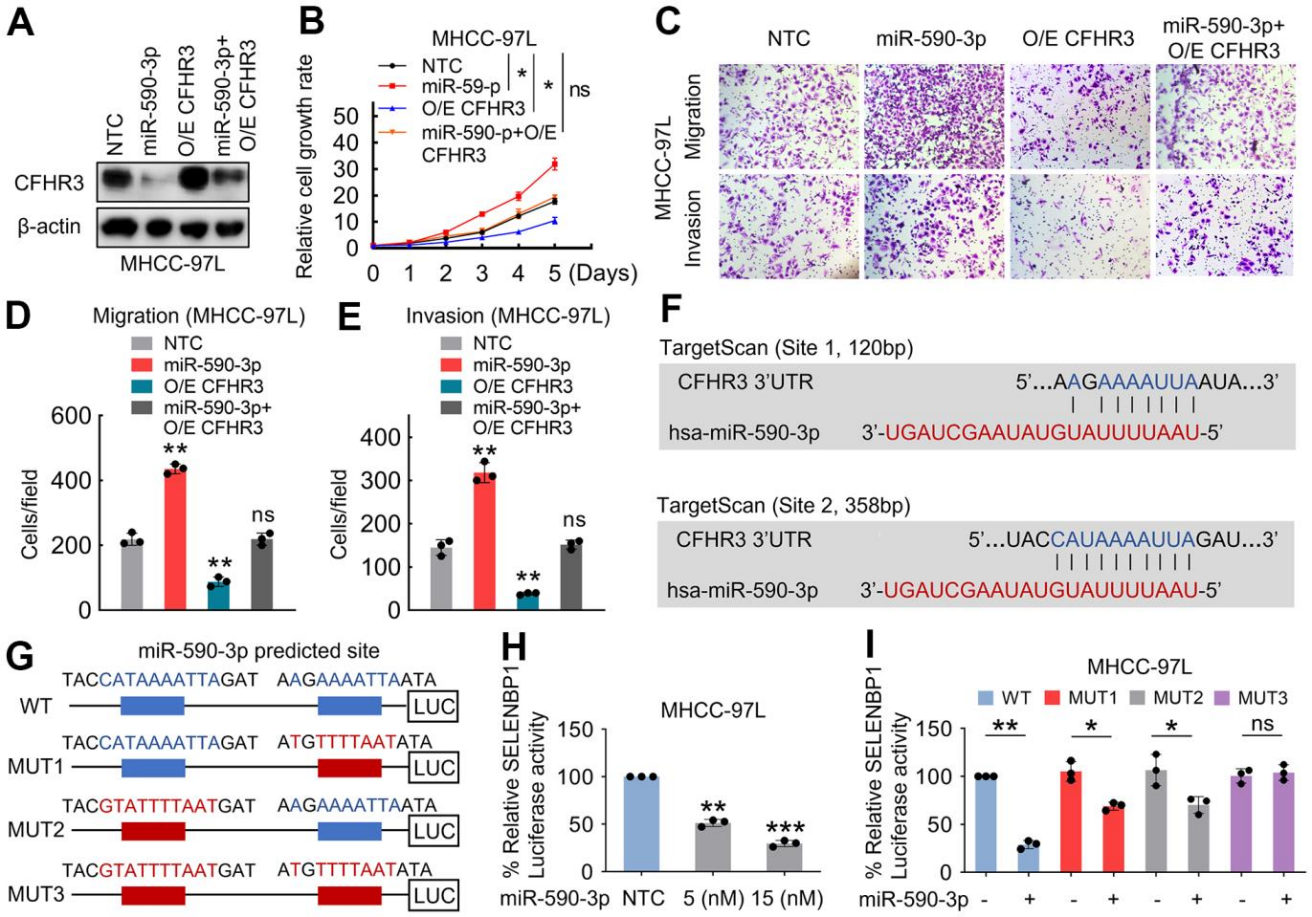
**Downregulation of CFHR3 is induced by miR-590-3p binding to the 3’ UTR of CFHR3.** (**A**–**E**) CFHR3-overexpressed MHCC-97L cells were treated with the indicated 15nM miR-590-3p precursor, and CFHR3 protein levels (**A**), cell growth (**B**), migration, and invasion (**C**–**E**) were determined. (**F**) Tow binding sites of miR-590-3p in the 3’ UTR of CFHR3 were predicted by the TargetScan programs. (**G**) A schematic diagram shows the miR-590-3p binding site in the 3’ UTR (wild-type and corresponding mutants) of CFHR3. (**H**) MHCC-97L cells were co-transfected with a CFHR3 3’ UTR reporter and the indicated concentrations of the miR-590-3p precursor, and the luciferase activities were measured (n=3). (**I**) MHCC-97L cells were co-transfected with miR-590-3p precursor and CFHR3 3’ UTR wild-type or indicated mutated reporter, and the luciferase activities were measured (n=3). (Remarks: * represents P < 0.05; ** represents P < 0.01; *** represents P < 0.001; Data are presented as mean ± SD).

To further explore whether CFHR3 is a leading effector in the regulation of malignant phenotypes of HCC cells induced by miR-590-3p, we transfected CFHR3 overexpression HCC cells with miR-590-3p precursor (Figure 4A). It showed that the expression of CFHR3 reversed the promotion of HCC cell growth, migration as well as invasion induced by miR-590-3p (Figure 4B–4E). Furthermore, two miR-590-3p binding sites were identified within the 3′ UTR of CFHR3 by using the target algorithm analyses (TargetScan) (Figure 4F). To validate that suppression of CFHR3 mRNA was owing to the induction by miR-590-3p, luciferase reporter constructs harboring CFHR3 3’ UTR (wild-type and corresponding mutants) were cloned (Figure 4G). miR-590-3p overexpression decreased luciferase activities (Figure 4H). Luciferase activities were not changed by overexpression while the 3’ UTR sequences for the seed region of miR-590-3p were mutated in the two complementary sites (Figure 4I). Taken together, these results demonstrate that the low level of CFHR3 is induced by the binding of miR-590-3p with the 3’ UTR of CFHR3.

### Downregulation of CFHR3 promotes phosphorylation of STAT3 and miR-590-3p/CFHR3/p-STAT3/p53 pathway is essential for HCC malignant phenotypes

**Figure 5:**
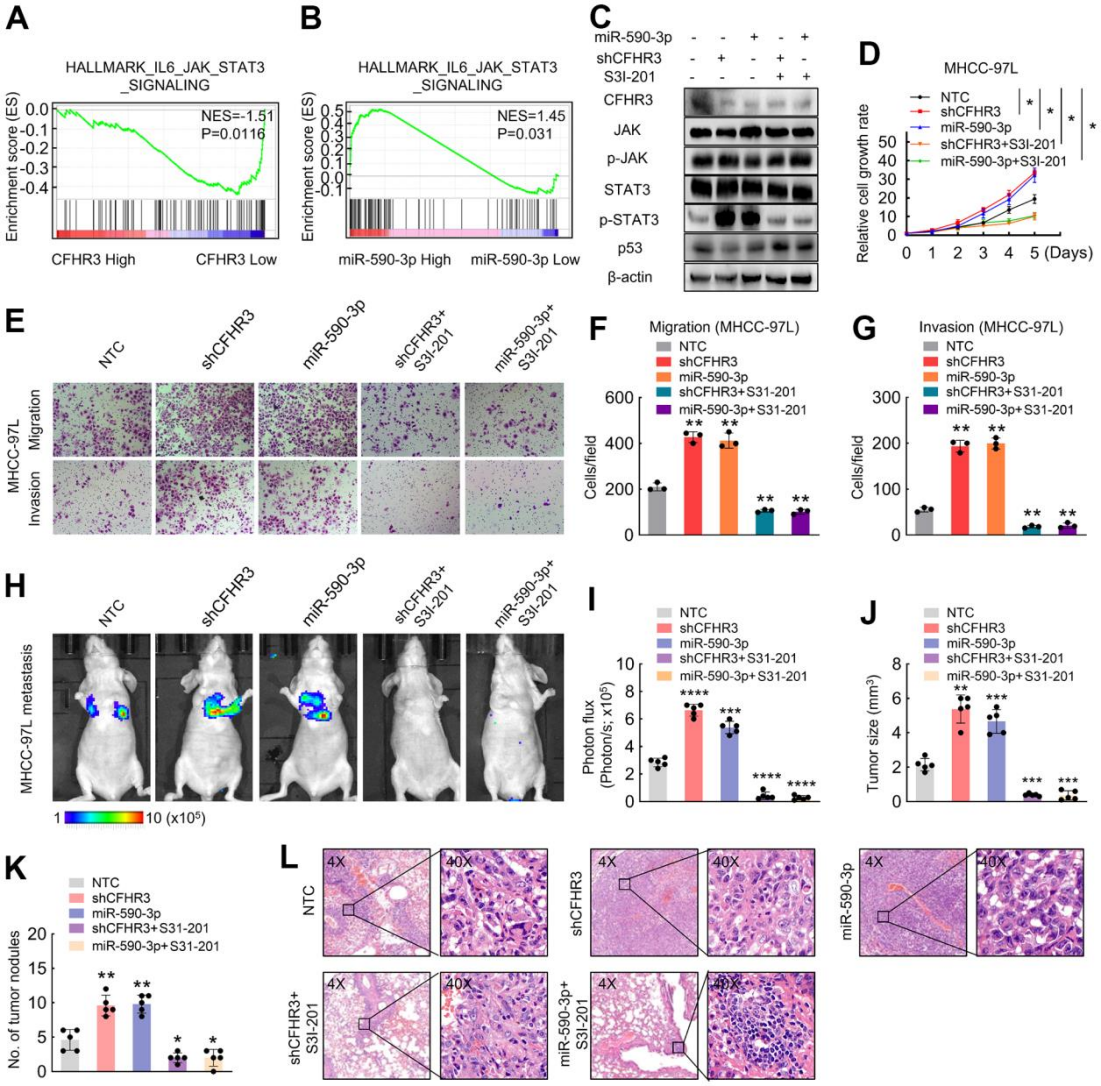
CFHR3 downregulation promotes phosphorylation of STAT3 and (**A**, **B**) GSEA plots show the enrichment of genes in response to JAK/STAT3 signaling between the HCC tissues with CFHR3^high^ and CFHR3^low^ (**A**), or between the HCC tissues with miR-590-3p^high^ and miR-590-3p^low^ (**B**). (**C**–**G**) MHCC-97L cells transfected with shCFHR3-RNAs or miR-590-3p precursor were treated with S3I-201 (50μM) or DMSO (control) for 8 hours, and the indicated protein levels (**C**), cell growth (**D**), migration and invasion (**E**–**G**) were investigated. (**H**, **I**) The indicated luciferase-labeled MHCC-97L cells (2 × 10^6^ cells/mouse) were injected into NOD-SCID mice (**H**); the luciferase activity was visualized 6 weeks post-transplantation (**I**, n = 5) (**J**, **K**) The tumor size (**J**) and number (**K**) of metastatic tumor nodules in mouse lung were observed and shown in a bar chart, respectively. (**L**) Representative IHC images of mouse metastatic tumors formed by MHCC-97L cells. (Remarks: * represents P < 0.05; ** represents P < 0.01; *** represents P < 0.001; **** represents P < 0.0001; Data are presented as mean ± SD).

**Figure 6:**
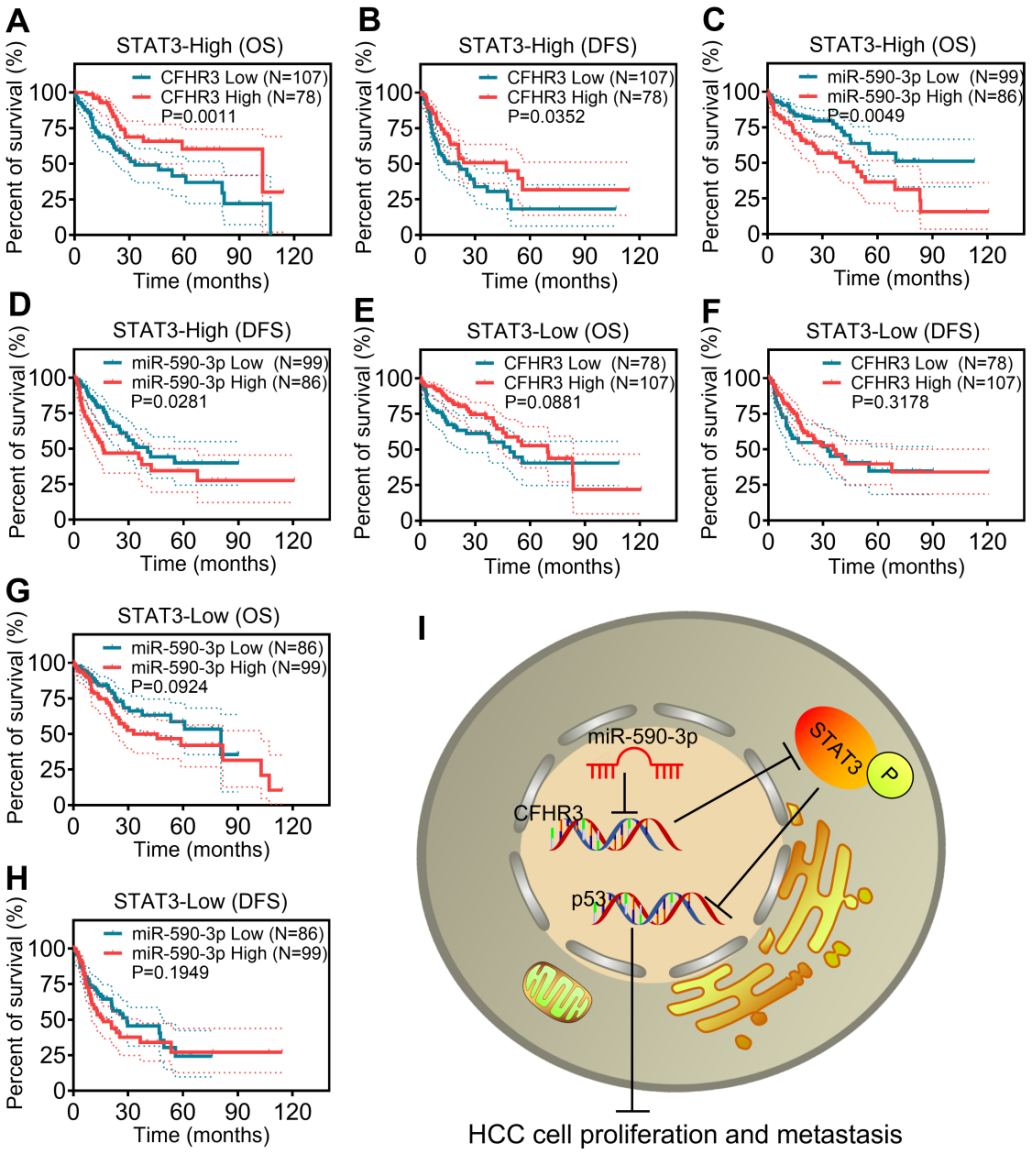
**miR-590-3p/CFHR3/p-STAT3/p53 pathway is required for HCC patient poor prognosis.** (**A**–**D**) The OS or DFS curves are based on CFHR3 transcript levels (**A**, **B**) or miR-590-3p levels (**C**, **D**) in HCC subgroups with STAT3 high expression. (**E**–**H**) The OS or DFS curves are based on CFHR3 transcript levels (**E**, **F**) and miR-590-3p levels (**G**, **H**) in HCC subgroups with STAT3 low expression. (**I**) A schematic overview of the signaling axis of miR-590-3p/CFHR3/p-STAT3/p53.

To further figure out regulation mechanisms in which CFHR3 downregulation promotes HCC cell malignant phenotypes, we performed GSEA analysis upon CFHR3 and miR-590-3p levels in TCGA-LIHC RNA sequencing data. It showed that genes related to JAK/STAT3 signaling showed enrichment in low-level-CFHR3 or high-level-miR-590-3p HCC tissues (Figure 5A, 5B), suggesting that the JAK/STAT3 pathway is likely to be potential downstream signaling upon the downregulation of CFHR3. Excessive activation of the JAK/STAT3 pathway was shown to play an essential role in the development and progression of different cancers [21–23]. Hence, we asked whether the HCC cell malignant phenotypes were enhanced by downregulation of CFHR3 is associated with activation of the JAK/STAT3 pathway. Interestingly, we found that reduction of CFHR3 induced by shRNAs of CFHR3 or miR-590-3p increased phosphorylation levels of STAT3, which was further suppressed by STAT3 inhibitor, S3I-201 (Figure 5C). In addition, the downregulation of CFHR3 did not alter the phosphorylation levels of JAK (Figure 5C). Previous studies have shown the normal growth and oncogenic signaling pathways in which phosphorylated STAT3 downregulates p53 gene expression [24–26]. As also shown in Figure 5C, p53 was altered reversely according to the status of phosphorylated STAT3, which means that phosphorylated STAT3 suppressed p53 in HCC cells. Furthermore, cell function assays showed that the promotional effects upon downregulation of CFHR3 induced by CFHR3 stable knockdown or miR-590-3p on HCC cell growth (Figure 5D), migration as well as invasion *in vitro* (Figure 5E–5G) as well as cell metastasis *in vivo* (Figure 5H–5L) were also attenuated by S3I-201. Moreover, p53 was decreased upon the increase of phosphorylated STAT3 due to the ectopic expression of STAT3, to antagonize the inhibition of HCC cell growth, migration as well as invasion induced by CFHR3 upregulation (Supplementary Figure 3A–3E). These data indicate that CFHR3 is specific and crucial for phosphorylation of STAT3 in HCC malignant phenotypes. Moreover, the survival analyses showed that in subgroups with high levels of STAT3, the HCC cases with CFHR3 downregulation or miR-590-3p upregulation presented worse OS or DFS (Figure 6A–6D). As for the subgroup with low expression of STAT3, low levels of CFHR3 or high levels of miR-590-3p did not exhibit the clinical significance in OS or DFS of HCC patients (Figure 6E–6H). Combined with our findings that miR-590-3p/CFHR3 regulated cell growth, migration, and invasion through STAT3 in HCC, here, we indicate that influence of CFHR3 on the prognosis of HCC patients is dependent on STAT3. Collectively, a miR-590-3p/CFHR3/p-STAT3/p53 signaling axis promoting HCC cell malignant phenotypes is elucidated (Figure 6I).

## DISCUSSION

In this paper, we found that CFHR3 downregulation was observed in HCC, which was essential for HCC patients’ poor prognoses. Despite CFHR3 low expression was detected in some certain cancers [7, 14, 27], the underlying molecular mechanisms of low CFHR3 expression in cancers are still largely unknown. Here, we revealed that levels of CFHR3 were negatively and significantly associated with miR-590-3p levels in HCC tissues. Several studies show that miR-590-3p is a decisive prognostic marker for cancers, including HCC, colorectal cancer, and non-small cell lung cancer [28–30]. In our further study, the inhibition of CFHR3 mRNA levels by miR-590-3p was figured out. We further revealed that CFHR3 downregulated mRNA levels were induced by the binding of miR-590-3p to the CFHR3’s 3’ UTR region. Accordingly, the identification of the miR-590-3p/CFHR3 axis illuminates how CFHR3 is inhibited in HCC.

Despite the molecular mechanism of CFHR3 mRNA inhibition being well elucidated, the molecular mechanism of how the downregulation of CFHR3 enhances HCC progression remains unclear. We further found that abilities of cell growth, migration as well as invasion of CFHR3-knockdown HCC cells were increased when compared with the NTC cells, suggesting that the downregulation of CFHR3 strengthens HCC cell growth and metastasis. Furthermore, the GSEA analyses imparted that JAK/STAT3 signaling pathway is likely to be a possible downstream responder of CFHR3. Similar to many other crucial signaling pathways, those pathways that are linked to cancer cell proliferation and metastasis are regulated by multitudinous positive as well as negative signaling that is initially induced by over-activation of JAK/STAT3 pathways [21, 31–33]. Here, we found that CFHR3 downregulation increased phosphorylation levels of STAT3 rather than these of JAK in HCC cells. More importantly, we further revealed that the downregulation of CFHR3 increased HCC cell growth and metastasis, which was suppressed by the STAT3 inhibitor, S3I-201. As known to us, phosphorylated STAT3 constitutes an essential pathway that regulates the signaling of miscellaneous biological activities including drug resistance, cell metastasis as well as cell proliferation [34, 35]. In our study, we confirm that the phosphorylated STAT3 induced by CFHR3 downregulation suppressed p53 expression to promote HCC progression. Therefore, these data suggested that CFHR3 is crucial for phosphorylated STAT3 in HCC malignant phenotype regulation. Generally, these findings elucidate that CFHR3 downregulation activates STAT3, promoting HCC cell proliferation and metastasis, indicating a novel role of CFHR3 in the regulation of cell aggressive phenotypes.

As long as the clinical significance in our research, it showed that the CFHR3 was an independent protective factor for OS and DFS of HCC patients. These findings highlighted a potentially vital performance of CFHR3 downregulation in HCC patients’ poor prognoses. More extraordinarily, we revealed that when the HCC patients were grouped by STAT3 mRNA levels, we were surprised to find that high levels of STAT3 no longer exhibited the promotion significance in HCC patients’ poor prognoses. Here, the clinical significance of the miR-590-3p-CFHR3-p-STAT3 axis in HCC was identified in this study.

Altogether, we find that CFHR3 functions as a protective protein for HCC patients. A novel function of CFHR3 in malignant phenotypes of HCC cells is now revealed, in which downregulation of CFHR3 enhances HCC cell growth and metastasis. Furthermore, the promotion effects of miR-590-3p/CFHR3/p-STAT3/p53 axis in HCC cell’s aggressive biological function are revealed. Therapeutic targeting the miR-590-3p/CFHR3/p-STAT3/p53 axis is a possible and effective strategy for the treatment of HCC.

## Supplementary Material

Supplementary Materials

Supplementary Figures

Supplementary Table 1
