# Correction for: The miR-590-3p/CFHR3/STAT3 signaling pathway promotes cell proliferation and metastasis in hepatocellular carcinoma

**DOI:** 10.18632/aging.204346

**Published:** 2022-10-15

**Authors:** Zhongzhong Wan, Xingrun Li, Xinru Luo, Bofan Wang, Xiang Zhou, Ao Chen

**Affiliations:** 1 Institute of Biology and Medicine, College of Life Science and Health, Wuhan University of Science and Technology, Wuhan 430081, Hubei Province, People’s Republic of China; 2 State Key Laboratory for Liver Research, The University of Hong Kong, Hong Kong, 0000, People’s Republic of China; 3 Department of Pathology, Li Ka Shing Faculty of Medicine, The University of Hong Kong, Hong Kong, 0000, People’s Republic of China

**This article has been corrected: **The authors requested that affiliations 2 and 3 of the last author, Dr. Ao Chen, be removed, as his position as a Hong Kong Scholar in the Department of Pathology, University of Hong Kong was only a short-term collaboration.

The correct authors list with the affiliations is listed below.

Zhongzhong Wan^1,*^, Xingrun Li^1,*^, Xinru Luo^1^, Bofan Wang^1^, Xiang Zhou^1^, Ao Chen^1^

^1^Institute of Biology and Medicine, College of Life Science and Health, Wuhan University of Science and Technology, Wuhan 430081, Hubei Province, People’s Republic of China

*Co-first author

