# Supplementary material for: The miR-590-3p/CFHR3/STAT3 signaling pathway promotes cell proliferation and metastasis in hepatocellular carcinoma
Source: Aging (Albany NY). 2022 Jul 18;14(14):5783–99. doi: 10.18632/aging.204178 (PMC9365569; doi:10.18632/aging.204178)
Supplement: Supplementary Materials [file aging-14-204178-s001.pdf]

SUPPLEMENTARY MATERIALS

shRNA sequences

shNTC:

Sense: 5’-CCGGAAGCTGACCCTGAAGTTCATTTCAAGAGAATGAACTTCAGGGTCAGCTTTTTTTTT-3’
Anti-sense: 5’-AATTAAAAAAAAGCTGACCCTGAAGTTCATTCTCTTGAAATGAACTTCAGGGTCAGCTT-3’

shCFHR3#1:

Sense: 5’-CCGGGGTCCTCTAGATGCAGTTAACTTCAAGAGAGTTAACTGCATCTAGAGGACCTTTTTT-3’
Anti-sense: 5’-AATTAAAAAAGGTCCTCTAGATGCAGTTAACTCTCTTGAAGTTAACTGCATCTAGAGGACC-3’

shCFHR3#2:

Sense: 5’-CCGGGCAGTTAACCAAATAGGGTCATTCAAGAGATGACCCTATTTGGTTAACTGCTTTTTT-3’
Anti-sense: 5’-AATTAAAAAAGCAGTTAACCAAATAGGGTCATCTCTTGAATGACCCTATTTGGTTAACTGC-3’

Vector construction primers

pLenti-CMV-blast-CFHR3:

Forward: 5’-GGGGATCCGCCACCatgttgttactaatcaatgtcat-3’
Reverse: 5’- GGctcgagttattcgcatctggggtattccac-3’
pGL3-Basic-CFHR3-3’ UTR (WT)-reporter:
Forward: 5’- ggtaccGGCAGCATTGTTACCCTAAATG-3’
Reverse: 5’- aagcttTAATTTATAAAGGAAAGAGG-3’
pGL3-Basic- CFHR3-3’ UTR (MUT1)-reporter:
Forward: 5’-TCTACTTTATTTCAATGTTTTAATATATAATAGTTTCAA-3’
Reverse: 5’-TTGAAACTATTATATATTAAAACATTGAAATAAAGTAGA-3’
pGL3-Basic- CFHR3-3’ UTR (MUT2)-reporter:
Forward: 5’-GGGAAGTAATAATACGTATTTTAATGATATTAAAATTGTA-3’
Reverse: 5’-TACAATTTTAATATCATTAAAATACGTATTATTACTTCCC-3’

miR-590-3p detected primers:

Forward: 5’-AAGGAGCUUACAAUCUAGCUGGG-3’
Reverse: 5’-CAGCUAGAUUGUAAGCUCCUUUU-3’
