# Supplementary material for: The miR-590-3p/CFHR3/STAT3 signaling pathway promotes cell proliferation and metastasis in hepatocellular carcinoma
Source: Aging (Albany NY). 2022 Jul 18;14(14):5783–99. doi: 10.18632/aging.204178 (PMC9365569; doi:10.18632/aging.204178)
Supplement: Supplementary Figures [file aging-14-204178-s002.pdf]

SUPPLEMENTARY FIGURES

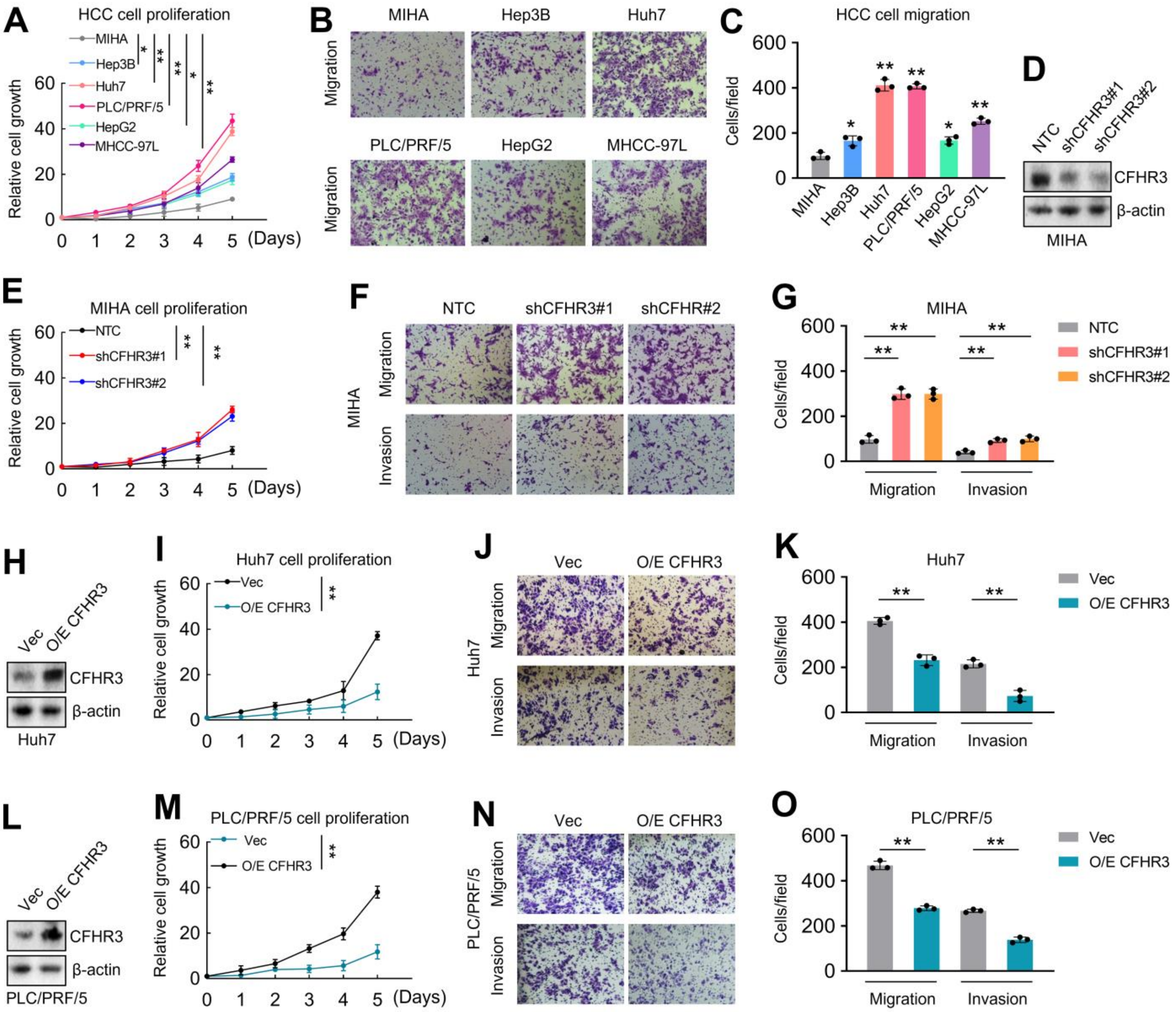

Supplementary Figure 1. Related to Figure 2. (**A**) The cell growth abilities of difference five HCC cell lines and the MIHA cell were determined (N=3). (**B**, **C**) The migration abilities of difference five HCC cell lines and the MIHA cell were determined (N=3). (**D**) CFHR3 knockdown efficiency was validated by western blot in MIHA cells. (**E**) Cell growth of CFHR3-knockdown MIHA cells and negative control (NTC) cells were determined (N=3). (**F**, **G**) Migration and invasion of CFHR3-knockdown MIHA cells and NTC cells were determined (N=3). (**H**) CFHR3 overexpression was validated by western blot in Huh7 cells. (**I**, **J**) Cell growth (**I**), migration and invasion (**J**, **K**) of CFHR3-overexpression Huh7 cells and Vector control (Vec) cells were determined (N=3). (**L**) CFHR3 overexpression was validated by western blot in PLC/PRF/5 cells. (**M**–**O**) Cell growth (**M**), migration and invasion (**N**, **O**) of CFHR3-overexpression PLC/PRF/5 cells and Vec cells were determined (N=3). (Remarks: * represents $P < 0.05$; ** represents $P < 0.01$; Data are presented as mean ± SD).

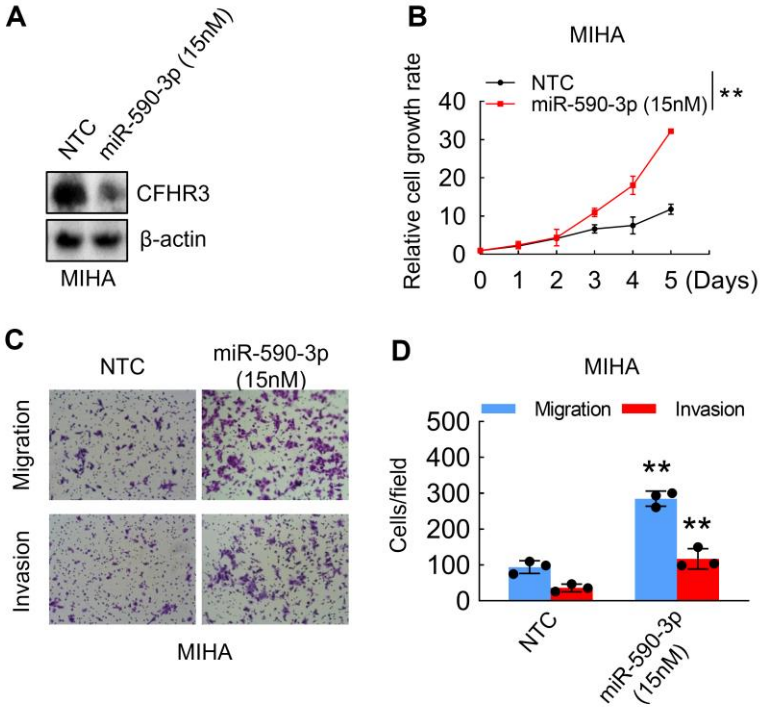

Supplementary Figure 2. Related to Figure 3. (**A**–**D**) MIHA cells were transfected with 15nM miR-590-3p precursor, and CFHR3 protein levels (**A**), cell growth (**B**), migration and invasion (**C**, **D**) were determined (N=3). (Remarks: * represents P < 0.05; ** represents P < 0.01; Data are presented as mean ± SD).

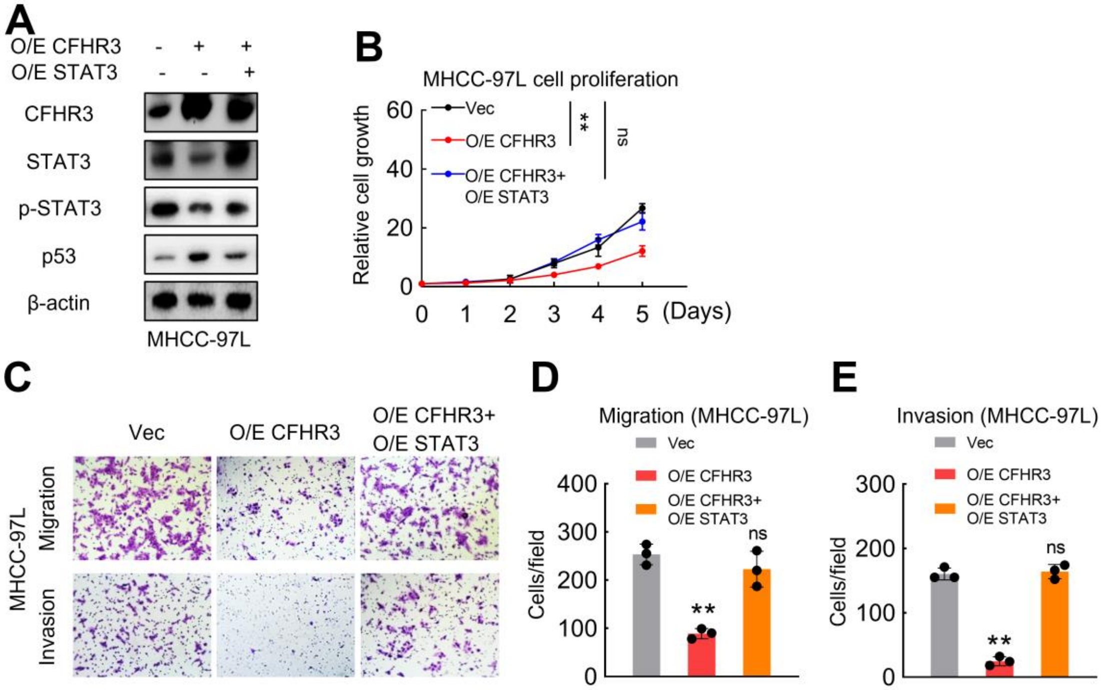

Supplementary Figure 3. Related to Figure 5. (**A**–**E**) CFHR3-overexpressed MHCC-97L cells were overexpressed with STAT3, and the indicated protein levels (**A**), cell growth (**B**), migration and invasion (**C**–**E**) were determined (N=3). (Remarks: * represents P < 0.05; ** represents P < 0.01; Data are presented as mean ± SD).
