# Supplementary material for: The miR-590-3p/CFHR3/STAT3 signaling pathway promotes cell proliferation and metastasis in hepatocellular carcinoma
Source: Aging (Albany NY). 2022 Jul 18;14(14):5783–99. doi: 10.18632/aging.204178 (PMC9365569; doi:10.18632/aging.204178)
Supplement: Supplementary Table 1 [file aging-14-204178-s003.pdf]

SUPPLEMENTARY TABLE

Supplementary Table 1. Correlations between CFHR3 transcript levels and clinicopathological features in 370 HCC cases of TCGA database.

Clinical features	Groups	All cases	CFHR3		X^2	P-value*
			Low	High		
Age	<60 Y	168	91	77	2.137	0.144
	>=60 Y	202	94	108		
Gender	Female	122	65	57	0.783	0.376
	Male	248	120	128		
AFP	<400 μg/L	213	89	124	7.263	0.007
	>=400 μg/L	64	39	25		
Fibrosis	No	75	46	29	0.944	0.331
	Yes	136	74	62		
Histological grade	G1-2	233	105	128	4.942	0.026
	G3-4	133	76	57		
Tumor size	T1-2	273	122	151	13.951	<0.001
	T3-4	95	63	32		
Recurrence	No	172	76	96	4.318	0.038
	Yes	143	80	63		
TNM stage	I-II	255	113	142	12.541	<0.001
	III-IV	91	61	31		

*Pearson Chi-square test.
